# Selective laser sintering of inkjet-printed silver nanoparticle inks on paper substrates to achieve highly conductive patterns

**DOI:** 10.1038/s41598-018-28684-4

**Published:** 2018-07-10

**Authors:** Enkeleda Balliu, Henrik Andersson, Magnus Engholm, Thomas Öhlund, Hans-Erik Nilsson, Håkan Olin

**Affiliations:** 1 Mid Sweden University, Department of Electronics Design, Sundsvall, 851 70 Sweden; 2 Mid Sweden University, Department of Natural Sciences, Sundsvall, 851 70 Sweden; 3 Mid Sweden University, Faculty of Science, Technology and Media, Sundsvall, 851 70 Sweden

## Abstract

Development of cost-effective and environmentally friendly manufacturing methods will enable important advances for the production of large-scale flexible electronics. Laser processing has shown to be a promising candidate that offers a fast and non-destructive way to produce highly conductive patterns on flexible substrates such as plastics. However, an emerging option with a lower environmental impact is instead the use of cellulose-based flexible substrates, such as paper. In this work we investigate the use of laser sintering of silver nanoparticle inks, which were inkjet-printed on three different types of paper. Patterns with a high conductivity could be manufactured where a special care was taken to prevent the substrates from damage by the intense laser light. We found that the best results was obtained for a photopaper, with a conductivity of 1.63 ∗ 10^7^ S/m corresponding to nearly 26% of the bulk silver conductivity. In addition, we demonstrate laser sintering to fabricate a fully functional near field communication tag printed on a photopaper. Our results can have an important bearing for the development of cost-effective and environmentally friendly production methods for flexible electronics on a large scale.

## Introduction

Printed electronics on flexible substrates has gained significant attention due to an increasing interest for everyday life applications such as flexible displays^1,2^, energy harvesting and storage (solar cells and super capacitors)^3,4^, as advanced medical sensors (lab on a chip)^5,6^ or similar. The aim is not to compete with regular electronics but rather to introduce a new class of devices that aims at large area applications that uses flexible and environmentally friendly substrates and materials. To succeed, the manufacturing processes need to be suitable for large area manufacturing in a roll-to-roll process. For most printed electronic applications, a highly conductive material in contacts and tracks is required in order to minimize the resistive losses. Conductive polymer inks generally have too low conductivity to fulfill the requirements^7^, which narrows down the choice to either conductive flake inks or nanoparticle (NP) inks. NP inks have the benefit of having a much higher conductance. This is because the NPs can be sintered together in a post process step whereas flake inks only rely on the physical contact between the flakes that are kept together with a binder material. NP inks are used mostly for ink-jet printing due to low ink waste, as nano particle inks generally have a high cost. The most commonly used metal for NP inks is silver (Ag)^8^ due to the good conductance and low oxidation. The metal NPs usually are coated with a polymer in order to avoid agglomeration in the ink dispersion. This polymer coating leads to a low conductivity and requires some type of post-processing to obtain a high conductivity. Some of the available post-processing techniques to sinter the AgNPs and increase the conductivity include oven-^9^, electrical-^10^, flash lamp-^11–13^, chemical-^14–16^ and laser sintering^17–22^.

Commonly used substrates for printed electronics are polyimide^23^, polyethylene terephthalate (PET)^24^ and other flexible plastic substrates^25–27^. An emerging option is the use of cellulose-based substrates, hereafter referred to as paper substrates. The interest in using paper as a substrate for printed electronics have increased considerably, partly because of the environmental aspect, but also because of the desire to integrate electronic functionality into paper-based products^28–31^ and for production of printed electronics on a large scale on paper substrates. However, many of the mentioned sintering techniques are not appropriate for large scale roll-to-roll manufacturing or compatible with paper substrates. The main reasons are the temperature sensitivity of the paper and the high speeds used in roll-to-roll (R2R) manufacturing, where the usefulness of traditional sintering techniques is limited.

The use of paper substrates for AgNP inkjet inks generally requires the use of suitable coatings. The main function of the coating layer is to provide a smooth surface to facilitate a continuous AgNP film formation. The coating layer is also important to provide an absorbent layer to quickly remove the ink solvent^32–34^. Few studies have been conducted on sintering of inkjet-printed AgNP films on paper substrates^13,14^. Although, the reported research work on laser sintering have, to our knowledge, included only a limited number of paper substrates. Therefore, in this work we investigate laser sintering of inkjet-printed AgNP patterns on different paper substrates as a method to create highly conductive patterns, with minimal impact on the paper substrate. Furthermore, the applied method is scalable and compatible with the standard methods used within R2R manufacturing. In addition to a photopaper, we include a lightweight coated (LWC) paper and cardboard (CB), since both of these paper types are extensively used within the packaging and graphics industry for large scale production.

Our investigation shows that highly conductive patterns can be formed on all of the selected paper substrates and the best results, with a conductivity of nearly 26 % compared to the bulk conductivity of silver, was reached on the photopaper substrate. In addition, we also demonstrate laser sintering for the fabrication of a fully functional near-field communication (NFC) tag.

## Methods

### Selected substrates and nanoparticle inks

The properties of the selected substrates to investigate are listed in Table 1, where the content of the paper coatings were previously characterized by FTIR^32^. Substrates P1–P5 are inkjet photopapers and have special absorption coatings with very high absorption rate. The CB and LWC substrates are not commonly used for inkjet printing and have a much lower absorption rate than P1–P5. The silver content of the inks are shown in Table 2.

**Table 1:** The substrates coating content, weight and relative solvent absorption rate.

Name	Substrate	Coating	Weight (*g*/*m*^2^)	Absorption rate
P1	Canon PT-101	AlO(OH)	300	High
P2	Epson Premium Glossy	SiO_2_	255	High
P3	Epson Glossy	AlO(OH)	225	High
P4	Brother	SiO_2_	260	High
P5	HP Advanced	SiO_2_ and AlO(OH)	250	High
CB	Invercote T Cardboard (Iggesund)	CaCO_3_/Clay	280	Low
LWC	Lightweight Coated (SCA)	CaCO_3_/Clay	60	Low

**Table 2:** The silver content and viscosity of the inks used.

Denomination	Ink	Silver contents (wt%)
Ink1	DGP-40LT-15C (Advanced Nano Products)	31
Ink2	CCI-300 (Cabot Corporation)	20

Laser sintering of Ag films on all papers (P1–P5, LWC, CB) using 100 mm/s scanning speed, revealed that all the photopapers (P1–P5) had a similar Ag film conductivity after sintering, while the LWC and CB substrates had a much lower conductivity. Considering these initial results we chose to only include the P1 photopaper, the LWC and the CB substrates for the rest of this study. Further, it was noted that the difference in conductivity when using Ink1 or Ink2 closely followed the silver content in the inks (Table 2). Considering that the inks were similar after compensating for the silver content, only Ink1 was used for the rest of the study.

### Preparation of the samples

**Figure 1:**
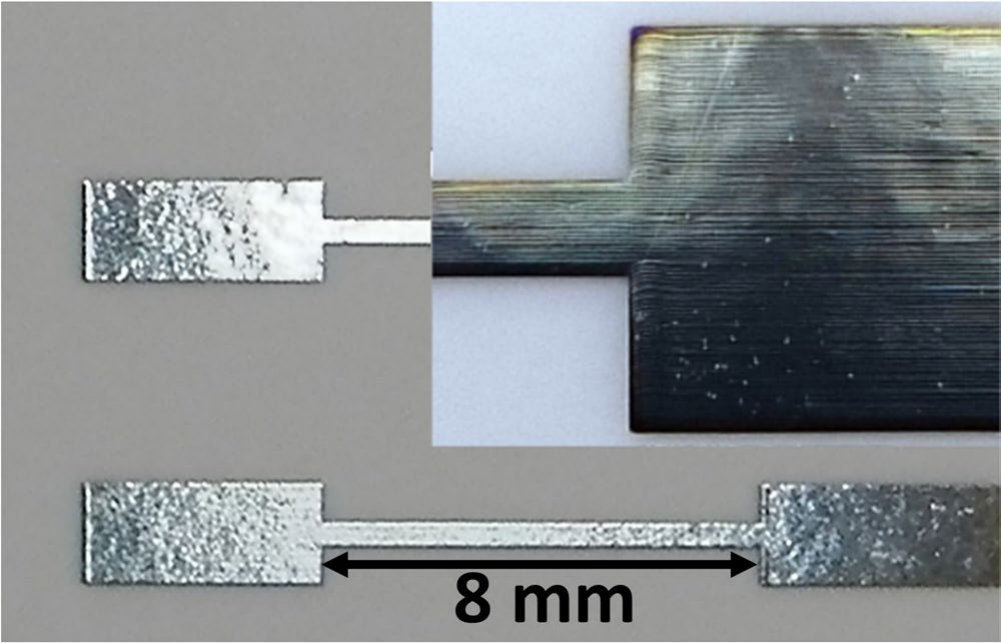
Inkjet test structures consisting of a 500 *μm* wide and 8 mm long line between contact pads. A magnified view is shown in the inset.

**Figure 2:**
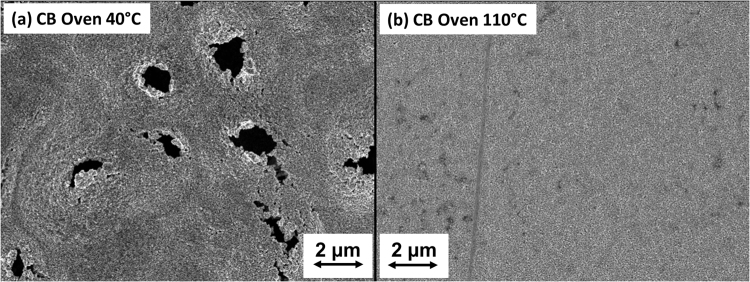
SEM images of laser processed CB samples preheated at different temperatures, 40 °C (**a**) and 110 °C (**b**). Severe damage were observed on samples preheated at 40 °C after the laser processing. Similar damage were also observed on the other paper substrates (P1–P5 and light weight coated paper (LWC) (not shown).

The printing was performed by using a Dimatix 2831 piezoelectric inkjet printer (Fujifilm, USA), with a 10 pL Dimatix 11610 cartridge. The substrates were kept in position by a vacuum plate with a temperature of 30 °C. The printed patterns consisted of lines (length 8 mm, width 500 *μm*) between contact pads, as displayed in Fig. 1. During our initial investigation, it was observed that the Ag films were damaged during the laser sintering. It is likely that the damage is due to the rapid heating, leading to evaporation of remaining solvent in the films. This is consistent with previous observations and conclusions of similar phenomena using flash sintering^35^. The observed damage was also found to be different for different samples, leading to the conclusion that there is a varying amount of solvent left after printing. Therefore, to obtain samples with the same starting characteristics it was determined to dry all samples in a convection oven. By inspection of differently pre-heated samples, see Fig. 2, a temperature of 110 °C during 20 minutes were chosen. The oven preheating process can be substituted with a low power laser heating process in a roll to roll process, however in our case, the oven preheating provides more unified starting characteristics considering the large sample diversity investigated.

Resistivity measurements were performed by the 4-wire resistance method using an HP 3440 multimeter. The electrical conductivity was calculated by the known equation *ρ* = *L*/(*R* ∗ *W* ∗ *t*) where L is the length, R the measured resistance, W the width and t the thickness of the sample. The thickness used to calculate conductivity was measured by using Scanning Electron Microscope (SEM).

### Laser sintering

The laser sintering was carried out by using a galvanometric scanning mirror system with a continuous wave (CW) fiber laser operating at 1064 nm with a spot size of 60 *μ*m and an intensity up to 250 kW/cm^2^. The printed samples were positioned on an aluminium plate and sintered by scanning the beam in a left-right-left raster pattern over the sample. The scanning speeds were 30, 100, 300, 1000 and 3000 mm/s and varying laser intensities were used. The morphology of the samples was examined by using a high resolution SEM (Zeiss Merlin). SEM characterisation was made of Ag films on CB and photopaper substrates, directly as printed, after oven sintering in 110 °C and after additional laser sintering. The coalescence of the NPs are dependent on the temperature increase in the material. The temperature of the NPs is dependent on the laser intensity, exposure time as well as the heat dissipation.

## Results

**Figure 3:**
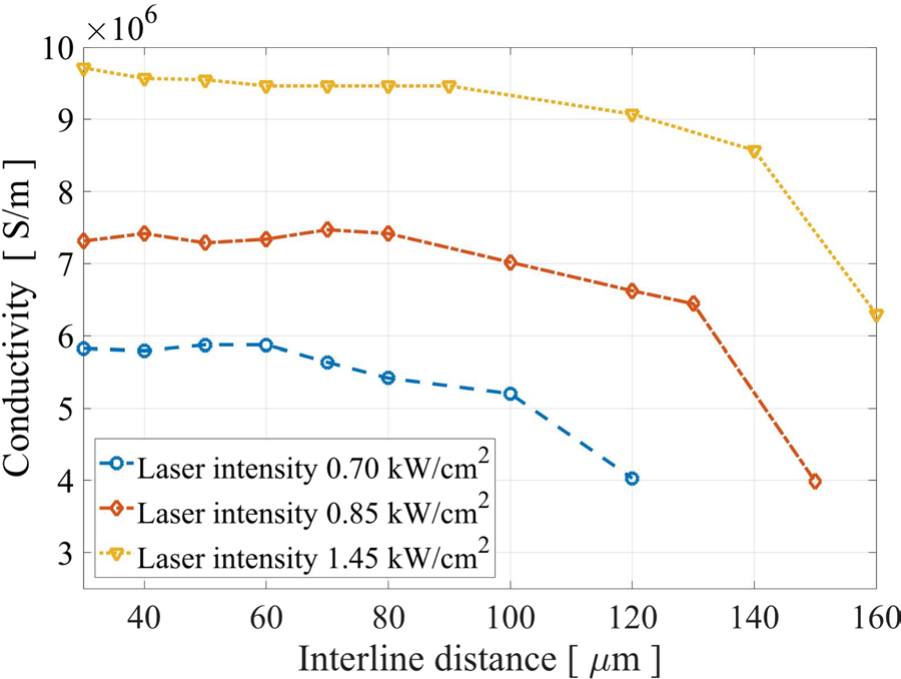
Conductivity as a function of the interline distance for different laser intensity. The conductivity decreases when the interline distance exceeds 60 *μ*m.

**Figure 4:**
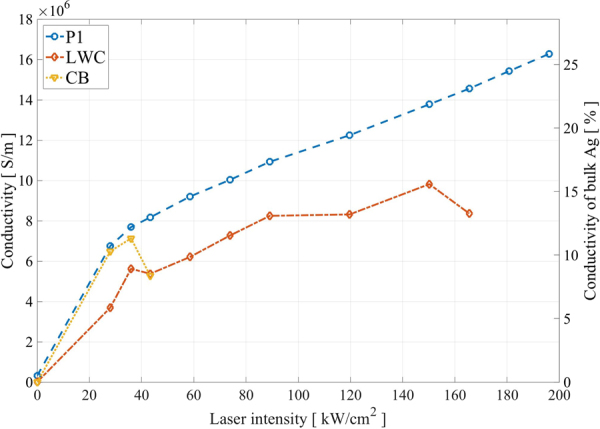
Conductivity as a function of laser intensity for Canon PT-101 P1 substrate (blue), light weight coated (LWC) paper (red) and cardboard (CB) (yellow) substrate at a scanning speed of 100 mm/s. The decrease on conductivity in the case of CB and LWC was because of burning of the paper substrate occurred.

**Figure 5:**
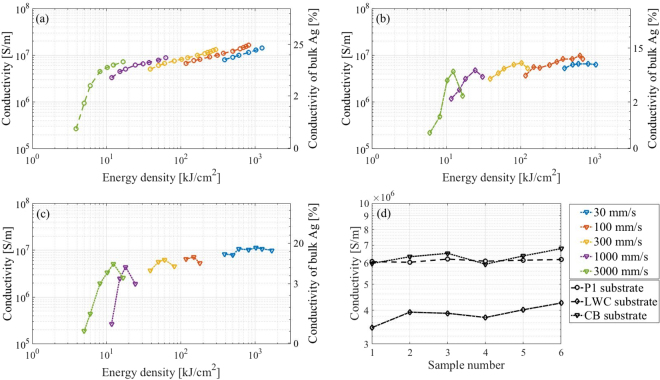
(**a**–**c**) Conductivity as a function of laser energy density for different laser scanning speeds. A decrease in speed results in an increase in energy density. For light weight coated (LWC) paper and cardboard (CB) substrate: The decrease on conductivity in the case of 30, 100 and 300 mm/s was because of the burning of the substrate. In the case of 1000 and 3000 mm/s, the decrease of conductivity was because of the delamination of the Ag films from the paper substrate. (**d**) Conductivity values of 6 samples for each substrate, which were laser processed at a scanning speed of 100 mm/s and an energy density of 244 kJ/cm^2^.

An important parameter of the laser sintering process is the distance between the laser line scans. During the laser sintering the laser beam will affect also some area outside of the spot due to the heat spreading. Therefore, a suitable distance between the laser line scans should be determined (interline distance). By sintering samples using varying interline distance and a fixed scanning speed of 300 mm/s, it was noticed that the conductivity decreased significantly when the interline distance was much larger than 60 *μ*m, the interline distance can be increase if increasing the laser intensity Fig. 3. Therefore, the interline distance was set to 60 *μ*m to ensure that no areas were left un-sintered, while still avoiding an excessive overlap. It was observed that there is a scanning speed threshold under which the substrate will be burnt, see Fig. 4. Moreover, different substrates have different resilience towards burning. This is most likely caused by the difference in the coatings, where P1 substrate has very thick coating in the range of 50 *μ*m, while the CB and LWC have much thinner coatings. Likewise, there was a threshold above which the delamination of the Ag films occurred, see Fig. 5(b,c). The delamination threshold was different for each substrate, where the LWC and CB had the lowest delamination thresholds. It is most likely that the delamination occurs because of the difference in thermal expansion between the heated silver NPs and the paper substrate. The lower conductivity observed in the case of CB and LWC is most likely due to damage in the Ag films, which was much more prevalent with those substrates according to SEM analysis. It is possible that the film damage is an effect of the lower solvent absorption rate of the CB and LWC substrates compared to the instant-drying P1–P5. This is because a lower absorption rate increases the likelihood of remaining solvent in the AgNP film, which can be very quickly evaporated by the rapid laser heating, damaging the film in the process.

**Figure 6:**
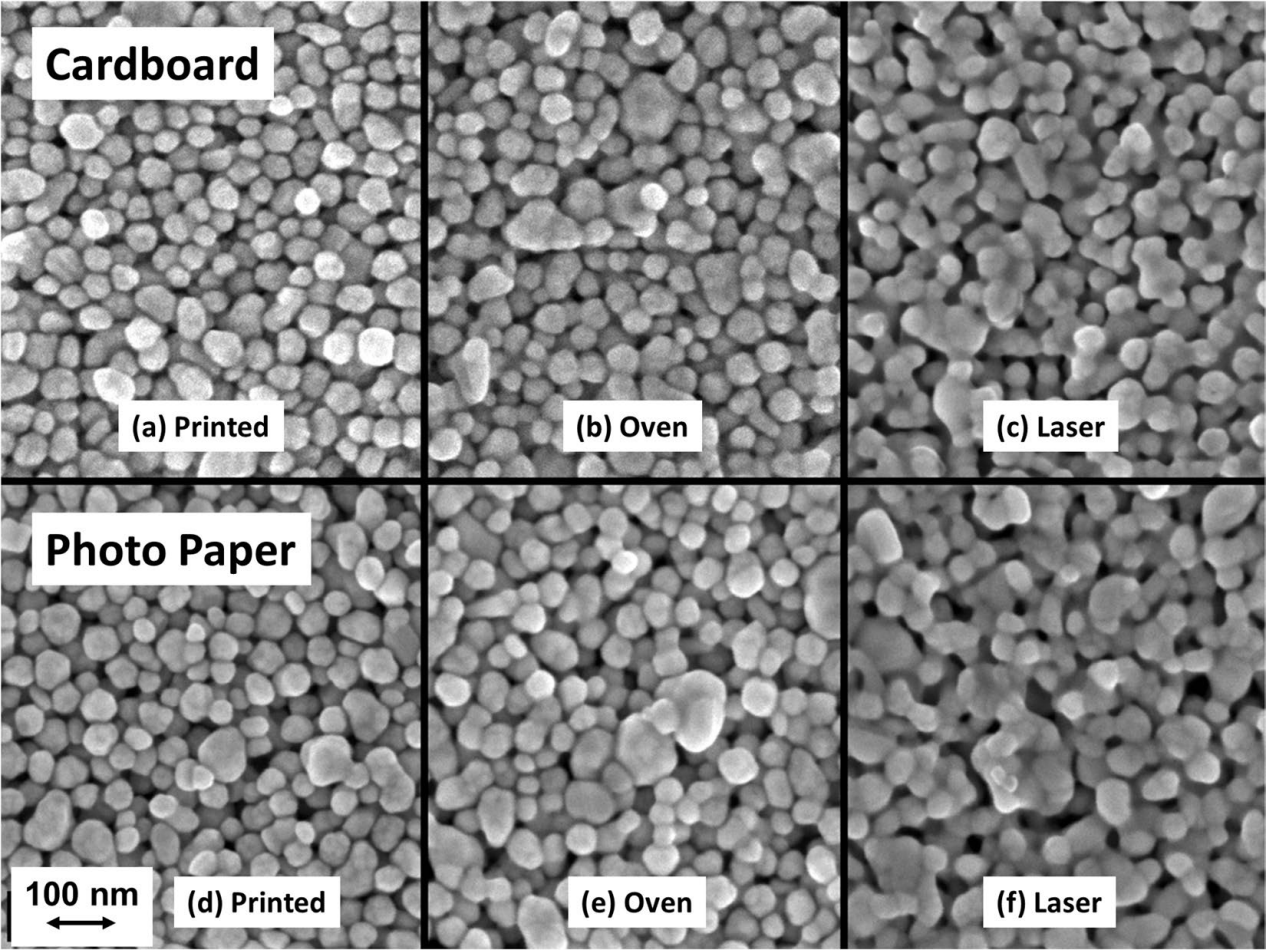
SEM images showing the AgNP coalescence for different processing conditions. Top left to right: Cardboard (CB); not processed, dried in oven at 110 °C in 20 min, additional laser processing. Bottom left to right: photopaper (P1); not processed, dried in oven at 110 °C in 20 min, additional laser processing.

From SEM analysis, it can be seen that the NPs have coalesced slightly during oven sintering compared to the as-printed samples, but to a much larger degree when laser-processed (as shown in Fig. 6). As expected, using a higher laser intensity, or a slower scanning speed, results in an increased conductivity due to a larger energy input into the sample. For the lowest scanning speed, 30 mm/s, burning was the primary failure mechanism on all substrates (burning was also the primary failure on LWC at 100 mm/s). For all the higher scanning speeds, such as 1000 mm/ and 3000 mm/s, delamination was the primary failure mechanism.

The conductivity was calculated using a thickness of 850 ± 150 *nm*, which was obtained by SEM measurements. This thickness is consistent with previous thickness measurements performed on the same ink printed on the same photopaper^28,36^. The cross section of the line was approximated to a rectangle using the measured thickness and width. It has previously been shown by the authors that such an estimate is on the conservative side as it will slightly overestimate the cross section, resulting in a slightly lower conductivity. The conductivity of the samples on the three substrates for different laser energy density and scanning speeds are presented in Fig. 5(a–c). It can be observed that the largest rate of conductivity increase happens in the low intensity range. This is expected as the first stage of sintering involves the removal of the solvent and the polymer protective shells, after which the NPs start to coalesce. After this initial stage, the conductivity increase will be slower due to the gradual densification and also possibly an increase in the reflectance of the AgNP film. The increased reflectance implies that a larger intensity is needed to further heat the film. This can be seen as a negative feedback mechanism, which helps to avoid overheating and film damage. This negative feedback mechanism has previously been identified as one of the advantages with using light for the sintering of metal NP films, when compared with other sintering methods^8,36^.

The conductivity measurement variation is shown in Fig. 5(d), where 6 samples were laser processed at a scanning speed of 100 mm/s and an energy density of 244 kJ/cm^2^. A typical standard deviation of the conductivity value for P1, CB and LWC are 6.6 ∗ 10^4^, 3.2 ∗ 10^5^ and 2.7 ∗ 10^5^ respectively, which correspond to 1.1%, 6.9% and 4.3% respectively. As expected the standard error mean is higher in the case of CB and LWC compared to P1 substrate.

**Figure 7:**
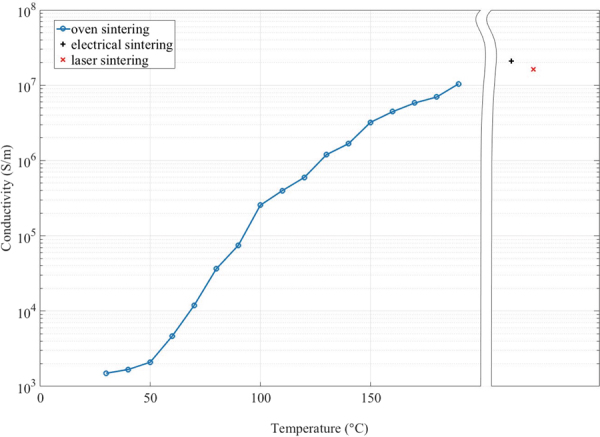
Comparison of the maximum conductivity reached for oven, electrical- and laser sintering methods.

As a comparison to laser sintering, sintering in a convection oven and electrical sintering were performed on samples printed on P1, see Fig. 7. To the electrical and laser sintering processes, the same preheating parameters were applied. The oven sintering was performed in steps of 10 °C from 30 °C to 190 °C, which was the upper limit for the photopaper. At this temperature the paper showed severe yellowing and the silver became very brittle and was not practical to use. The threshold before visible yellowing of the paper started was 150 °C.

The electrical sintering was performed in two steps using a constant current source, where the lowest resistance was achieved by applying 0.5 A followed by 0.9 A for approximately one second each. This resulted in the lowest resistance that could be achieved without destroying the sample, but also resulted in a very brittle silver trace that were impractical for use. The results are plotted in Fig. 7 in comparison with the best result obtained for the laser sintering, which can be seen to match the result of the electrical sintering, but without the problem of making the samples brittle.

### Application example: near-field communication tag

**Figure 8:**
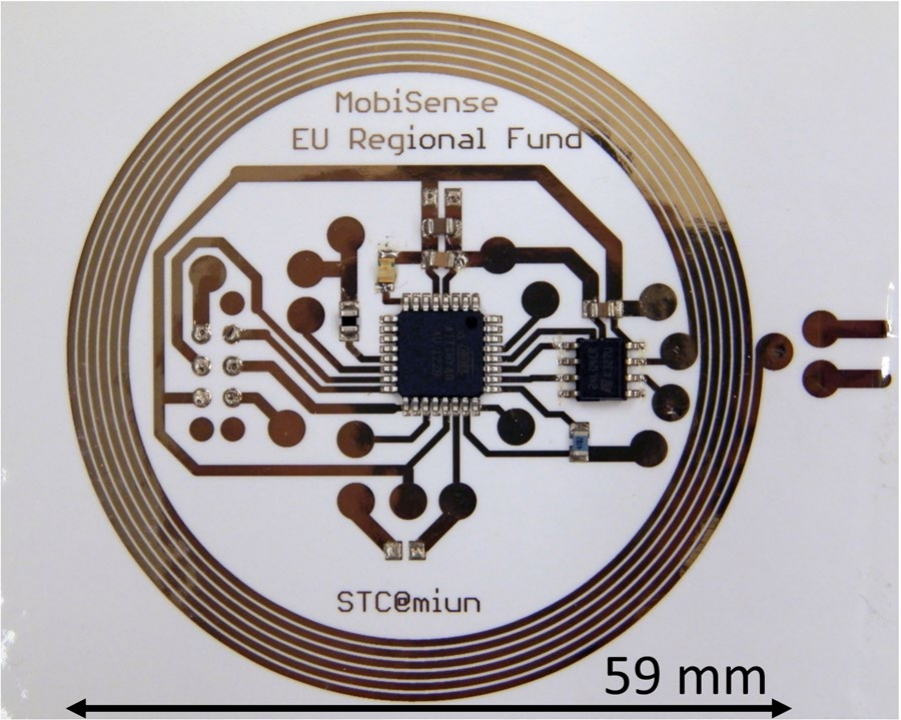
Complete and functioning hybrid NFC tag on a paper substrate. The antenna coil, circuit tracks, and contact pads are inkjet printed and laser sintered, while the microprocessor and NFC chip are soldered onto the printed contact pads.

Near-field communication (NFC) is a subset of radio frequency identification, which have recently increased in popularity due to the fact that a vast majority of smart phones now have NFC readers built in. One interesting new area is sensor-enabled NFC tags printed directly on packages or on labels. Such sensor tags can be used to measure a multitude of different parameters, such as humidity, temperature, or chemical contaminants in the air, depending on the particular sensor used. To demonstrate the advantages of laser sintering, we fabricated a fully functional NFC tag on photopaper (Fig. 8). The paper-based NFC tag uses an antenna coil that is 961 mm long with a 0.6 mm line width. A proper functionality of the tag requires a sufficiently low resistance of the coil, which in turn requires a very effective sintering process of the inkjet-printed coil. The antenna coil had a total resistance of 1.25 kΩ before sintering, which was lowered to 320 Ω after an initial run of laser sintering, and lowered again to 215 Ω after a second run. In comparison, using oven sintering at 150 °C which was the maximum temperature that did not damage the substrate, resulted in a coil resistance of more than 600 Ω, rendering the tag unusable for its intent (near-field communication with a smartphone to read a connected temperature sensor). More detail has been given in^37^.

## Discussion

Sintering of printed polymer capped nano-particles is a complex phenomenon, where the system consists of the silver nano-particles, polymer as well as remaining solvent. The size of the particles will influence the sintering time significantly according to Herrings scaling law^38^. The laser dwell time, the speed as well as the thermal properties of the AgNP ink and paper substrates will influence the sintering^39^. It is interesting to consider the conductivity as a function of energy density for different scanning speeds, as shown in Fig. 5. The different plots for the different scanning speeds is seen to not overlap although they have the same energy density but rather each decreasing scanning speed is seen to start at a lower conductivity value. This means that for the same deposited energy the resulting conductivity is higher for higher scanning speeds. For example, a scanning speed of 3000 mm/s gives a maximum conductivity of 7.28 ∗ 10^6^ S/m at 16.6 kJ/cm^2^ but for a scanning speed of 100 mm/s, to achieve the same conductivity requires an energy density of 180 kJ/cm^2^.

The explanation could be found in the heat dissipation in the sample. The heat dissipated through the Ag NP ink, the paper substrate and to the surrounding atmosphere is constant per unit time, meaning that if an amount of energy is deposited during a shorter time the dissipated heat during this time will be smaller and therefore the peak temperature will be higher. The type of paper substrate will influence the dissipation of heat, as the structure of the paper will have an effect on the thermal resistance of the papers. The photo paper have very thick coating layers compared to the LWC and CB, in the range of 50 *μ*m, that are designed to absorb the ink solvent and give a smoother surface for printing. The differences in coatings is also likely the reason that delamination and burning are observed for the LWC and CB while not for the photopaper as the NPs in the ink will attach better to the photopaper surface. The fast absorption of the solvent in the NP ink promotes a better adhesion and uniformity. Many sintering processes follow Arrhenius behaviour^40^, which states that the reaction rate is proportional to *e*^−1/*T*^ where T is the reaction temperature. Although we did not prove Arrhenius behaviour, we can conclude that using a laser with high power at a high speed is not only faster, but also more energy-efficient than using a laser with lower power scanned with a lower speed. However, there is a limit on the maximum usable intensity, since above a certain threshold, delamination or other damage will inevitably occur. It is most likely that the delamination occurs because of the difference in thermal expansion between the heated silver NPs and the paper substrate. In the case of burning, it is more likely that the lower laser scanning speed results in a more uniform heating of the silver and the substrate together due to heat spreading. With increased laser power, this leads to heating and damage of the substrate rather than to sintering of the NPs. Considering this, it is advantageous to use a higher power during a lower time to heat up the Ag NPSs, although below the power threshold for delamination.

We noted that the laser sintering on these substrates required pre-drying to avoid damage due to the rapid evaporation of ink solvent, see Fig. 2. The ink solvent is triethylene glycol monoethyl ether (TGEE) which has a boiling point of 256 °C, having a very slow evaporation in room-temperature. Pre-drying could be performed in a roll-to-roll process by passing the paper through a pre-heating section after the inkjet printing. The results and applicability of laser sintering are dependent on the paper coating, the laser process parameters such as scanning speed and intensity, as well as other factors such as the evaporation rate of the ink solvent and the pre-drying conditions. This study suggests that using processing parameters toward higher speed and higher intensity, combined with pre-drying, is a viable strategy to develop a R2R-compatible sintering platform for the production of large-area flexible electronics.

## Conclusion

Laser sintering of printed metal nanoparticles patterns has become of a great interest for the production of large scale flexible electronics. The laser sintering offers a fast and non-destructive process. A low environmental impact production of large area highly conductive film it is possible with the use of the paper substrate. Even though the high interest of large area paper based electronics, very few research group have worked on understanding the laser sintering process. In this work we investigated laser sintering of inkjet-printed AgNP inks on different paper substrates. Patterns with a high conductivity could be manufactured where a special care was taken to prevent the substrates from damage by the intense laser light. The best results were obtained when using a photopaper as substrate, with a conductivity of 1.63 ∗ 10^7^ S/m corresponding to nearly 26% of the bulk silver conductivity. This study suggests that the laser sintering is very dependent on the paper coating, therefore a preheating in oven of the paper substrate is needed. The preheating gives the possibility of a larger investigation where can be used different paper substrates. As a demonstration, we used laser sintering to fabricate a fully functional near-field communication tag on a photopaper, which was not possible using oven sintering. This study give the possibility to develop a R2R-compatible sintering platform for the production of large-area flexible electronics.
